# Demographic factors and correlates of trust in types of people and information sources: findings from the SAM-KAP Alabama youth survey on HIV prevention

**DOI:** 10.1186/s12875-025-03022-6

**Published:** 2025-10-31

**Authors:** Brittany A. Shelton, Ibrahim Yigit, Abigail Coulter, Danielle Trotter, Henna Budhwani

**Affiliations:** 1 Department of Public Health, University of Tennessee Knoxville, 1914 Andy Holt Ave, HPER 372, Knoxville, TN 37996 USA; 2 College of Nursing, Florida State University, Tallahassee, FL USA

**Keywords:** HIV, Adolescence, Prevention, Knowledge, Trust

## Abstract

**Background:**

Sexual and gender minority adolescents (SGM) experience higher rates of discrimination than heterosexual and cisgender peers, which may lead to adverse health behaviors, including condomless sex. Experienced discrimination and stigmatization may lead SGM adolescents to disproportionately seek information online around HIV risk and prevention. Understanding trusted sources of information among SGM adolescents is critical to addressing the HIV epidemic.

**Methods:**

Two hundred six SGM adolescents, with male sex assigned at birth, aged 14–17 years, were recruited in-person from an LGBTQ-supportive school in Alabama, online via social media, and via a youth center. Respondents reported trust in information about pre-exposure prophylaxis (PrEP) from different types of people and information sources using a Likert scale with higher scores indicating greater trust. We utilized multivariable linear regression models to estimate the relationship between social support, self-efficacy, internalized homophobia, depression, discrimination, HIV, STI, and HIV prevention knowledge, and trust in types of people and information sources.

**Results:**

White adolescents (*n* = 127) reported higher trust in HIV doctors (M: 4.83, SD: 0.92 vs. M: 4.72, SD: 0.81, *p* = .04) than Black adolescents (*n* = 63) who reported higher trust in religious leaders (M: 2.17, SD: 1.23 vs. M: 2.76, SD: 1.33, *p* = .002). Hispanic adolescents (*n* = 25) reported lower trust in straight friends/peers as compared to non-Hispanic peers (*n* = 167) (M: 2.42, SD: 1.16 vs. M: 3.19, SD: 1.10, *p* = .003). Social support and self-efficacy were significantly associated with trust in healthcare providers (β = 0.24, *p* = .013; β = 0.19, *p* = .040, respectively). Social support was associated with trust in family members (β = 0.23, *p* = .019). HIV prevention knowledge (β = 0.15, *p* = .046) and self-efficacy (β = 0.21, *p* = .010) were significantly associated with trust in Instagram. Self-efficacy was associated with trust in television commercials (β = 0.26, *p* = .009) and religious leaders (β = 0.20, *p* = .048).

**Conclusion:**

Social support and self-efficacy were salient correlates of trust. Self-efficacy may reflect the perceived ability to better care for oneself. Social support may lead SGM adolescents to self-select into healthcare from affirming providers, resulting in higher levels of trust in those providers. These findings indicate the potential value of bolstering social support and self-efficacy in preventative health interventions and may serve as a foundation for future intervention development to disseminate information about PrEP.

## Introduction

Adolescence is a critical stage of human development, noted as the life-stage in which individuals experience the most substantial change across multiple dimensions [1, 2]. During this time of growth, the influence of parents and guardians may wane as adolescents increasingly seek approval from their peers. The period of adolescence is also marked by increased risk-taking and sensation-seeking that, in tandem with seeking approval from peers, may predispose adolescents to engage in health-risk behaviors such as alcohol use and condomless sex [1]. These health-risk behaviors, in turn, may elevate their risk of sexually transmitted infections (STIs) and HIV acquisition. In the United States (US), adolescents and young adults accounted for 48.2% of incident STIs and 20% of incident HIV diagnoses in 2022 [3]. Moreover, the greatest burden of STIs and HIV is in the southern US where the requirements of sex education are not inclusive of the specific needs of sexual and gender minority (SGM) adolescents, potentially leading this population to seek information from sources outside of formal education settings. As formative health behaviors may develop during this period [4], ensuring adolescents have access to high quality health information from trusted sources may promote uptake of HIV prevention, specifically pre-exposure prophylaxis (PrEP), and consquently reduce their risk of HIV.

Disparities in the prevalence of STIs and HIV among adolescents are evident [3, 5–7], suggesting certain groups of adolescents are at elevated risk of these adverse health conditions. Specifically, adolescent SGM who were assigned male sex at birth, engage in higher rates of substance use and condomless sex that elevate their risk of HIV and STIs as compared to their cisgender and heterosexual peers. Meta-analyses suggest that, among SGM adolescents, 50% did not use a condom the last time they had sex [8]. Moreover, 32% engaged in substance use during their last sexual encounter [8, 9]. This pattern of health-risk behavior is particularly concerning as the predominant risk behavior associated with HIV acquisition in the US is male-to-male sexual contact (MMSC) [3]. As illustrated by the Minority Stress Model [10], typical health-risk behaviors among SGM adolescents may be more prevalent as they constitute a mechanism for coping with greater experiences of stigmatization and discrimination relative to their cisgender and heterosexual peers [11]. Moreover, social support or the lack thereof may influence endured stress and corresponding efforts to address stress levels [12]. This lack of social support may consequently lead SGM adolescents to increasingly affiliate and seek health information from peers or alternative information sources, such as social media. Consequently, greater understanding of where SGM adolescents seek information about PrEP is critical to ensure high quality health information is provided.

Where SGM adolescents obtain trusted information about HIV prevention, specifically PrEP, is unclear. Adolescents seek autonomy and independence that may result in greater desire to seek information on their own rather than relying upon parents and guardians. In a national study from 2005 to 2015, doctors were the most trusted source of health information for young adults [13]. However, social media’s role in distributing healthcare information and misinformation has become more pervasive as 98% of adolescents reported using social media within the past month [14]. With the increased adoption, integration, and utilization of smartphone technology in daily life, the opportunity for health information and disinformation is increasing. SGM adolescents may disproportionately seek health information from online sources due to discrimination, stigmatization, or lack of social support. Moreover, those SGM adolescents residing in rural areas may have limited access to affirming healthcare services or adequate social support equipped to meet their health education needs [15, 16]. For transgender and gender diverse (TGD) adolescents, the role of online information seeking may be even more salient as they may feel even more isolated as a result of discriminatory or stigmatizing experiences. Additionally, TGD adolescents report that their level of trust in differing information sources is influenced by the broader socio-political environment [17]. Thus, in this study, we aimed to examine who SGM adolescents in Alabama view as trusted sources of information on PrEP. Identifying trusted people and sources of information may permit better tailoring of health information to meet their unique needs for health education thereby reducing risk of HIV acquisition.

## Methods

### Parent study

The sample included SGM adolescents recruited in-person from an LGBTQ-supportive school, online via social media, and via a youth center through the “Survey of Adolescent MSM Knowledge and Preferences (SAM-KAP)” project, also called the “Alabama Youth Survey.” [18] To participate, individuals had to meet the following criteria: [1] be between the ages of 14 and 17 years [2], be assigned male sex at birth [3], express sexual partner preference of males, and [4] reside in Alabama. Participants completed a survey that assessed their knowledge of HIV and STIs, along with other measures such as internalized homophobia, depression, and demographic information. The survey took approximately twenty minutes to complete, and participants received a $35 incentive upon completion. Signed informed assent was collected from adolescents. The Institutional Review Board approved a waiver of parental consent; guardians were informed of the study with the option to have their child opt out via an informational document. The Florida State University Institutional Review Board approved this study (STUDY00003480), and all research was performed in accordance with IRB guidelines and regulations and the Declaration of Helsinki.

### Measures

#### Demographics and health behaviors

Demographics (age, race, ethnicity, and gender identity) were self-report. Similarly, HIV testing, sexual activity, and history of substance use were self-report. Individuals who affirmed recent HIV testing, sexual activity, and/or substance use were subsequently asked whether these activities or an STI diagnosis occurred in the past six months.

#### Trust in types of people

Trust in types of people related to PrEP information was assessed with a single question: “If you wanted to learn more about PrEP, please rank how much you’d trust information on PrEP from the following people.” The individuals listed included pediatricians, HIV doctors, nurses, pharmacists, parents or guardians, and siblings. The item was rated on a 5-point Likert scale, ranging from 1 (completely distrust) to 5 (completely trust), with higher scores indicating greater trust in that individual. In this study, using these categories of individuals, we created two scores for model analyses: trust in healthcare providers (i.e., pediatricians, HIV doctors, nurses, and pharmacists) and trust in family members (i.e., parents or guardians, and siblings), with mean scores calculated by averaging the corresponding items.

#### Trust in information sources

Trust in information sources to learn about PrEP was assessed with a single question: “If you wanted to learn more about PrEP, please rank how much you’d trust these information sources.” The sources included web search/google; online news source (e.g., New York Times, CNN, or FOX News); Tik Tok; Instagram; Twitter (X); Reddit; Discord; pamphlets in a clinic; pamphlets in a school; television commercials; radio commercials; straight friends/peers; and religious leaders, pastors, and preachers. The item was rated on a 5-point Likert scale ranging from 1 (completely distrust) to 5 (completely trust), where higher scores indicated greater trust in that information source. Mean scores were calculated by taking the mean of the items.

#### HIV knowledge

The HIV Knowledge Questionnaire (HIV-KQ-18) [19] was used to assess knowledge about HIV transmission, prevention, and misconceptions. It consists of 18 true-false items, with one point awarded for each correct answer and zero points for incorrect answers or responses of “don’t know” or “prefer not to answer.” Higher scores indicate greater knowledge related to HIV. Sample items are “Coughing and sneezing don’t spread HIV” and “People who have been infected with HIV quickly show serious signs of being infected.” In this study, Cronbach’s alpha was 0.76.

#### STI knowledge

The STI Knowledge Scale [20] was used to assess knowledge about STIs, including symptoms, transmission, prevention, and treatment. It consists of 27 true-false items, with one point awarded for each correct answer and zero points for incorrect answers or responses of “don’t know” or “prefer not to answer.” Higher scores indicate greater knowledge about STIs. Sample items include “There is a vaccine that can protect a person from getting hepatitis B” and “Human Papillomavirus (HPV) can cause HIV.” Cronbach’s alpha was 0.82.

#### HIV prevention knowledge

Knowledge about PrEP for HIV prevention was measured using the Knowledge PrEP Scale [21], which consists of 10 true-false items. One point awarded for each correct answer, and zero points were given for incorrect answers or responses of “don’t know” or “prefer not to answer.” Sample items include “PrEP is a daily pill you can take to reduce your risk of becoming infected with HIV” and “You should not use PrEP if you don’t know your HIV status +.” Higher scores indicated greater knowledge related to PrEP. Cronbach’s alpha as 0.83.

#### Internalized homophobia

The Revised Internalized Homophobia Scale (IHP-R) [22] was used to measure internalized homophobia, referring to the negative attitudes and feelings that individuals who identify as gay, lesbian, or bisexual (GLB) may internalize about their own sexual orientation. It consists of five items rated on a 5-point Likert scale ranging from 1 (disagree strongly) to 5 (agree strongly), with higher scores indicating more negative self-attitudes. Sample items are “I wish I weren’t gay/bisexual” and “I have tried to stop being attracted to men in general.” Cronbach’s alpha was 0.88.

#### Everyday discrimination scale

The Everyday Discrimination Scale [23], consisting of nine items, was used to assess experiences of discrimination based on race, ethnicity, gender, age, or other characteristics. Items were rated on a 6-point Likert scale ranging from 1 (almost every day) to 6 (never), with higher scores indicating greater experiences of discrimination. Sample items include “You are called names or insulted” and “People act as if they’re better than you are.” Cronbach’s alpha was found to be 0.92.

#### Depression

The Patient Health Questionnaire-8 (PHQ-8) [23] was used to assess symptoms of depression. It consists of eight items (e.g., “Feeling down, depressed, or hopeless” and “Feeling tired or having little energy”) rated on a 4-point Likert scale ranging from 0 (not at all) to 3 (nearly every day), with higher scores indicating higher depressive symptoms. This version does not include questions on self-harm. In this study, Cronbach’s alpha was 0.91.

#### Social support

The 19-item MOS Social Support Survey [24] was used to measure the perceived availability of social support across several dimensions (i.e., emotional/informational, tangible, affectionate support, and positive social interaction). Items were rated on a 5-point Likert scale ranging from 1 (none of the time) to 5 (all of the time), with higher scores indicating higher perceived social support. In this study, Cronbach’s alpha was 0.96.

#### Self-efficacy

The General Self-efficacy Scale (GSE) [25], consisting of 10 items rated on a 4-point Likert scale (1 = not true at all, 4 = exactly true), was used to measure participants’ ability to handle and cope with a wide variety of challenging situations. Sample items include “I can usually handle whatever comes my way” and “I can always manage to solve difficult problems if I try hard enough.” Higher scores indicate higher self-efficacy. In this study, Cronbach’s alpha was 0.89.

### Data analysis

Descriptive statistics were calculated for the sample. Independent samples t-tests were conducted to examine variations in the study variables across different groups (i.e., race, gender, and ethnicity). Given the exploratory nature of this study, we applied a Bonferroni-Hochberg correction to all p-values to account for inflated risk of a Type I error attributable to multiple testing [26]. Pearson’s correlation analysis and linear regression analyses were used to determine predictors of trust variables (i.e., trust in types of people and trust sources). In these regression analyses, we first tested simple linear regression models to estimate the relationship between each associate (e.g., internalized homophobia) and each outcome variable (e.g., trust in healthcare providers or trust in online news source), adjusting for race, gender, and ethnicity (adjusted models). Additionally, using all significant associates for each outcome from previous models, we tested multivariate liner regression models, adjusting for the control variables. All analyses were cross-sectional, with mean scores used for each variable. The data were analyzed using SPSS (version 29).

## Results

### Descriptive statistics

Descriptive statistics for the sample are presented in Table 1. The sample included 206 adolescent SGM individuals, with a mean age of 16.21 (standard deviation, SD = 0.88). Of these, 76.7 (*n* = 165) identified as men, with the majority identifying as White (60.8%, *N* = 127) and Non-Hispanic, Latino, or Spanish (87%, *n* = 167). Nearly 70% (*n* = 143) reported never having been tested for HIV. Among those reporting sexual activity in the last six months, only 53.2% (*n* = 58) reported consistent condom use.

**Table 1:** Descriptive statistics for the whole sample (*N* = 206)

Variables	*N* (%)
Gender identity*	
Man	165(76.7)
Transgender woman	13(6.0)
Other**	37 (17.1)
Race*	
White	127(60.8)
Black or African American	63(30.1)
Other ^A^	19 (9.1)
Ethnicity	
Hispanic, Latino, or Spanish	25(13)
Ever been tested for HIV?	
Yes	55 (26.7)
No	143 (69.4)
Prefer not to answer	1 (0.5)
Sex in the last 6 months?	
Yes	110 (53.4)
No	89 (43.2)
Prefer not to answer	6 (2.9)
Used condoms consistently in the last 6 months? ***	
Yes	58 (53.2)
No	51 (47.7)
STI in the last six months? ***	
Yes	8 (7.3)
No	101 (92.7)
Injectable substances in the last 6 months?	
Yes	18 (8.7)
No	176 (85.4)
Prefer not to answer	3 (1.5)

^*A*^ *Includes Asian, Native Hawaiian or Other Pacific Islander, American Indian or Alaskan Native*

*May select multiple answers

**Includes Gender Non-Binary/Genderqueer/Gender Nonconforming,* Agender*,* and Bigender*

***Among those reporting sexual activity in the last six months

### Comparisons for trust variables by race, gender, and ethnicity

To examine the differences in trust variables across race, gender, and ethnicity groups, we conducted a series of independent samples t-tests. As shown in Table 2, for trust in HIV doctors, there was a significant difference between White (mean, M = 4.83, SD = 0.53) and Black adolescents (M = 4.62, SD = 0.81, *p* =.037), with White adolescents reporting higher trust in HIV doctors. Black adolescents reported significantly higher trust in politicians compared to White adolescents (M = 2.26, SD = 1.29 vs. M = 1.81, SD = 1.07, *p* =.011). For trust in straight friends/peers, a significant difference was also observed between White (M = 3.13, SD = 1.06) and Black adolescents (M = 3.46, SD = 0.98, *p* =.040), with Black adolescents reporting higher trust in straight friends/peers. Black adolescents, compared to their White counterparts, also reported significantly higher trust in religious leaders, pastors, and preachers (M = 2.76, SD = 1.33 vs. M = 2.17, SD = 1.23, *p* =.002). Lastly, trust in an online news source was significantly higher among Black adolescents (M = 3.32, SD = 1.27) compared to White adolescents (M = 2.80, SD = 1.31, *p* =.008).

**Table 2:** Comparisons for trust types and information sources by race, gender, and ethnicity

Variables	Race		p*	Gender		p*	Ethnicity		p*
	White (*N* = 120)	Black (*N* = 63)		Male (*N* = 165)	Trans + GNC (*N* = 41)		Non-Hispanic (*N* = 167)	Hispanic (*N* = 25)	
	M(SD)	M(SD)		M(SD)	M(SD)		M(SD)	M(SD)	
Trust in types of people									
* Pediatrician*	4.12(0.92)	4.13(1.16)	0.935	4.05(1.07)	4.43(0.68)	0.041	4.12(1.02)	4.26(1.13)	0.542
* HIV Doctor*	4.83(0.53)	4.62(0.81)	0.037	4.69(0.73)	4.97(0.16)	0.027	4.76(0.66)	4.73(0.68)	0.903
* Nurses*	4.22(0.68)	4.38(0.81)	0.122	4.26(0.76)	4.47(0.56)	0.036	4.27(0.74)	4.56(0.58)	0.078
* Pharmacists*	4.10(0.88)	4.24(0.98)	0.385	4.15(0.97)	4.23(0.74)	0.774	4.10(0.96)	4.52(0.66)	0.042
* Teachers*	3.18(1.05)	3.37(1.14)	0.268	3.20(1.17)	3.03(0.97)	0.541	3.21(1.10)	2.90(1.22)	0.222
* Counselors and Psychologists*	3.59(1.06)	3.62(1.08)	0.890	3.56(1.16)	3.54(0.85)	0.914	3.56(1.10)	3.47(1.16)	0.752
* Politicians*	1.81(1.07)	2.26(1.29)	0.011	2.00(1.21)	1.53(0.77)	0.069	1.95(1.19)	1.47(0.74)	0.057
* Parents or Guardians*	3.55(1.20)	3.49(1.12)	0.869	3.65(1.10)	3.00(1.39)	**0.009**	3.51(1.20)	3.59(1.14)	0.874
* Siblings*	3.30(1.17)	3.24(1.18)	0.900	3.30(1.24)	2.91(1.16)	0.326	3.27(1.19)	2.85(1.42)	0.106
* Gay and Bi- Friends/Peers*	3.98(0.91)	4.15(0.85)	0.274	4.05(0.94)	4.14(0.59)	0.612	4.10(0.85)	3.85(1.10)	0.244
* Straight Friends/Peers*	3.13(1.06)	3.46(0.98)	0.040	3.06(1.17)	3.19(0.92)	0.326	3.19(1.10)	2.42(1.16)	**0.003**
* Religious Leader*,* Pastors*,* and Preachers*	2.17(1.23)	2.76(1.33)	**0.002**	2.40(1.33)	1.86(1.15)	0.078	2.34(1.31)	1.76(1.13)	0.036
Trust in information sources									
* Web Search/Google*	3.75(0.99)	3.91(1.19)	0.346	3.72(1.15)	3.84(0.79)	0.472	3.79(1.08)	3.40(1.18)	0.120
* Online News Source*	2.80(1.31)	3.32(1.27)	**0.008**	3.03(1.34)	2.35(1.19)	0.029	2.95(1.36)	2.52(1.16)	0.123
* Tik Tok*	2.53(1.23)	2.84(1.42)	0.111	2.65(1.32)	2.24(1.30)	0.173	2.58(1.32)	2.63(1.39)	0.958
* Instagram*	2.64(1.27)	2.68(1.36)	0.748	2.73(1.34)	2.08(1.06)	0.017	2.65(1.34)	2.33(1.11)	0.264
* Reddit*	2.73(1.34)	2.89(1.43)	0.399	2.76(1.38)	2.59(1.36)	0.716	2.79(1.39)	2.38(1.32)	0.185
* Discord*	2.54(1.35)	2.56(1.25)	0.877	2.51(1.36)	2.45(1.15)	0.972	2.51(1.30)	2.13(1.24)	0.181
* Pamphlets in a Clinic*	4.35(0.68)	4.25(0.86)	0.416	4.25(0.84)	4.46(0.56)	0.169	4.32(0.78)	4.14(0.85)	0.335
* Pamphlets in a School*	3.72(0.94)	3.72(0.86)	0.982	3.71(0.97)	3.57(1.04)	0.499	3.68(0.98)	3.66(1.06)	0.933
* Television Commercials*	3.30(1.09)	3.29(1.07)	0.745	3.35(1.19)	2.92(0.98)	0.097	3.36(1.11)	2.76(1.26)	0.018
* Radio Commercials*	3.22(1.03)	3.21(1.01)	0.680	3.20(1.15)	2.97(0.80)	0.481	3.22(1.05)	2.85(1.19)	0.120
* Straight friends/peers*	3.18(1.01)	3.11(1.04)	0.156	3.08(1.10)	3.00(0.98)	0.982	3.15(1.04)	2.57(1.12)	0.014
* Religious Leader*,* Pastors*,* and Preachers*	2.50(1.34)	2.40(1.38)	0.089	2.53(1.37)	1.89(1.09)	0.041	2.48(1.6)	1.71(0.90)	**0.008**

*p-values bolded indicate statistical significance after the Bonferroni-Hochberg correction for multiple testing

Next, transgender + GNC adolescents (M = 4.97, SD = 0.16) also reported significantly higher trust in HIV doctors compared to cisgender male adolescents (M = 4.69, SD = 0.73; *p* =.041). Cisgender male adolescents (M = 3.65, SD = 1.10) reported significantly higher trust in parents or guardians compared to transgender + GNC adolescents (M = 3.00, SD = 1.39, *p* =.009). As for trust in information sources, male adolescents (M = 3.03, SD = 1.34) reported significantly higher trust in an online news source compared to transgender + GNC adolescents (M = 2.35, SD = 1.19, *p* =.029). Trust in Instagram was also significantly higher among male adolescents (M = 2.73, SD = 1.34) compared to transgender + GNC adolescents (M = 2.08, SD = 1.06, *p* =.017). Finally, male adolescents (M = 2.53, SD = 1.37) reported significantly higher trust in religious leaders, pastors, and preachers compared to transgender + GNC adolescents (M = 1.89, SD = 1.09, *p* =.041).

In terms of ethnicity, non-Hispanic adolescents (M = 3.19, SD = 1.10) reported significantly higher trust in straight friends/peers compared to Hispanic adolescents (M = 2.42, SD = 1.16); *p* =.003). Regarding trust in information sources, non-Hispanic adolescents (M = 3.36, SD = 1.11) reported significantly higher trust in television commercials compared to Hispanic adolescents (M = 2.76, SD = 1.26, *p* =.018). Non-Hispanic adolescents (M = 3.15, SD = 1.04) also reported significantly higher trust in straight friends/peers compared to Hispanic adolescents (M = 2.57, SD = 1.12, *p* =.003). Lastly, trust in religious leaders, pastors, and preachers was also significantly higher among non-Hispanic adolescents (M = 2.48, SD = 1.60) compared to Hispanic adolescents (M = 1.71, SD = 0.90, *p* =.008).

### Predictors of trust in types of people and information sources

We first examined the correlation coefficients between the study variables via Pearson’s correlation analyses (see Table 3). Results showed that HIV knowledge, STI knowledge, social support, and self-efficacy were significantly and positively correlated with trust in healthcare providers (*r* =.18, *p* =.012; *r* =.14, *p* =.049; *r* =.33, *p* <.001; *r* =.25, *p* <.001, respectively). Internalized homophobia was also significantly and negatively correlated with trust in healthcare providers (*r*=-.21, *p* =.004). Next, social support and self-efficacy were significantly and positively correlated with trust in family members (*r* =.27, *p* <.001; *r* =.29, *p* <.001, respectively), while depression and discrimination were significantly and negatively correlated with trust in family members (*r*=-.21, *p* =.003; *r*=-.20, *p* =.007, respectively).

**Table 3:** Correlation coefficients among study variables

Variables	HIV knowledge	STI knowledge	HIV prevention knowledge	Internalized homophobia	Depression	Discrimination	Social support	Self-efficacy
Trust in types of people								
Trust in healthcare providers	0.18*	0.14*	0.04	− 0.21**	− 0.10	− 0.13	0.33**	0.25**
Trust in family members	− 0.01	− 0.01	0.01	− 0.09	− 0.21**	− 0.20**	0.27**	0.29**
Trust in information sources								
Online news source	0.09	0.08	0.07	− 0.14	− 0.22**	− 0.19**	0.23**	0.26**
Instagram	0.01	0.03	0.15*	− 0.03	− 0.11	− 0.12	0.15*	0.15*
Television commercials	0.19**	0.13	0.23**	− 0.27**	− 0.21**	− 0.19*	0.33**	0.31**
* Straight friends/peers*	0.06	0.02	0.02	− 0.06	− 0.14	− 0.18*	0.08	0.17*
* Religious leader*,* pastors*,* and preachers*	0.00	0.00	0.11	0.01	− 0.22**	− 0.19*	0.15*	0.26**

*p <.05

**p <.01

Next, adjusted linear regression analyses with race, gender, and ethnicity (see Table 4), created based on significant correlations, indicated that HIV knowledge, STI knowledge, social support, self-efficacy, and internalized homophobia were significantly associated with trust in healthcare providers (β = 0.23, *p* =.003; β = 0.17, *p* =.025; β = 0.40, *p* <.001; β = 0.37, *p* <.001; β=−0.23, *p* =.003, respectively). Trust in family members was also significantly predicted by social support (β = 0.32, *p* <.001), self-efficacy (β = 0.27, *p* =.001), depression (β=−0.26, *p* =.002), and discrimination (β=−0.20, *p* =.013). In the adjusted multivariable regression model, where all significant predictors were entered simultaneously (see Table 4), social support and self-efficacy were significant predictors of trust in healthcare providers (β = 0.24, *p* =.013; β = 0.19, *p* =.040, respectively), while only social support was a significant predictor of trust in family members (β = 0.23, *p* =.019).

**Table 4:** Adjusted and multivariate adjusted regression models for trust in type of people and trust information sources

Predictors	Adjusted simple regression models^A^		Multivariate adjusted regression models^A^	
	β	*p*	β	*p*
Trust in types of people				
Outcome: Trust in healthcare providers				
HIV knowledge	0.23	**0.003**	0.14	0.156
STI knowledge	0.17	**0.025**	0.01	0.874
Internalized homophobia	− 0.23	**0.003**	− 0.07	0.394
Social support	0.40	**< 0.001**	0.24	**0.013**
Self-efficacy	0.37	**< 0.001**	0.19	**0.040**
R-square	--	**--**	0.21	
F-value (*p*-value)	--	**--**	6.18	**< 0.001**
Outcome: Trust in family members				
Depression	− 0.26	**0.002**	− 0.13	0.227
Everyday discrimination	− 0.20	**0.013**	− 0.05	0.646
Social support	0.32	**< 0.001**	0.23	**0.019**
Self-efficacy	0.27	**0.001**	0.08	0.416
R-square	--	**--**	0.12	
F-value (*p*-value)	--	**--**	4.09	**< 0.001**
Trust in information sources				
Outcome: Trust in online news source				
Depression	− 0.27	**< 0.001**	− 0.20	0.063
Discrimination	− 0.16	**0.046**	0.05	0.645
Social support	0.29	**< 0.001**	0.14	0.150
Self-efficacy	0.32	**< 0.001**	0.19	0.051
R-square	--	**--**	0.18	
F-value (*p*-value)	--	**--**	5.67	**< 0.001**
Outcome: Trust in Instagram				
HIV prevention knowledge	0.19	**0.014**	0.15	**0.046**
Social support	0.15	0.059	-	-
Self-efficacy	0.24	**0.002**	0.21	**0.010**
R-square	--	**--**	0.12	
F-value (*p*-value)	--	**--**	5.10	**< 0.001**
Outcome: Trust in television commercials				
HIV knowledge	0.18	**0.022**	0.04	0.613
HIV prevention knowledge	0.21	**0.006**	0.09	0.272
Internalized homophobia	− 0.22	**0.007**	− 0.03	0.748
Depression	− 0.22	**0.008**	0.02	0.854
Discrimination	− 0.23	**0.004**	− 0.15	0.142
Social support	0.33	**< 0.001**	0.11	0.269
Self-efficacy	0.38	**< 0.001**	0.26	**0.009**
R-square	--	**--**	0.18	
F-value (*p*-value)	--	**--**	4.31	**< 0.001**
Outcome: Trust in straight friends/peers				
Self-efficacy	0.18	**0.034**	-	-
R-square	--	**--**	--	--
F-value (*p*-value)	--	**--**	--	--
Outcome: Trust in religious leader, pastors, and preachers.				
Depression	− 0.26	**0.001**	− 0.19	0.076
Discrimination	− 0.16	**0.043**	0.00	0.985
Social support	0.20	**0.012**	0.02	0.813
Self-efficacy	0.27	**< 0.001**	0.20	**0.048**
R-square	--	**--**	0.13	
F-value (*p*-value)	--	**--**	4.39	**< 0.001**

^A^Adjusted for race, gender, and ethnicity.

As for trust information sources^1^, as shown in Table 3, trust in an online news source was correlated with depression (*r*=-.22, *p* =.002), discrimination (*r*=-.19, *p* =.009), social support (*r* =.23, *p* =.002), and self-efficacy (*r* =.26, *p* <.001). Trust in Instagram was significantly correlated with HIV prevention knowledge (*r* =.15, *p* =.033), social support (*r* =.15, *p* =.045), and self-efficacy (*r* =.15, *p* =.043). Trust in television commercials was significantly correlated with all study variables (*p* <.05) except for STI knowledge (*r* =.13, *p* =.079). Trust in straight friends/peers was significantly correlated with discrimination (*r*=-.18, *p* =.016) and self-efficacy (*r* =.17, *p* =.018). Lastly, trust in religious leaders, pastors, and preachers was significantly correlated with depression (*r*=-.22, *p* =.002), discrimination (*r*=-.19, *p* =.012), social support (*r* =.15, *p* =.037), and self-efficacy (*r* =.26, *p* <.001).

Adjusted linear regression analyses (see Table 4) indicated that depression (β=−0.27, *p* <.001), discrimination (β=−0.16, *p* =.046), social support (β = 0.29, *p* <.001), and self-efficacy (β = 0.32, *p* <.001) were significantly associated with trust in an online news source. HIV prevention knowledge (β = 0.19 *p* =.014) and self-efficacy (β = 0.24, *p* =.002) were significantly associated with trust in Instagram. HIV knowledge, HIV prevention knowledge, internalized homophobia, depression, discrimination, social support, and self-efficacy were significantly associated with trust in television commercials (*p* <.05). Only self-efficacy was significantly associated with trust in straight friends/peers (β = 0.18, *p* =.034). Lastly, depression (β=−0.26, *p* =.001), discrimination (β=−0.16, *p* =.043), social support (β = 0.20, *p* =.012), and self-efficacy (β = 0.27, *p* <.001) were significantly associated with trust in religious leaders, pastors, and preachers.

Next, we conducted a series of adjusted multivariable regression models for each trust source outcome (except for trust in straight friends/peers, which had a significant association with only self-efficacy). As shown in Table 4, results indicated non-significant associations between trust in an online news source and depression, discrimination, social support, and self-efficacy. Both HIV prevention knowledge (β = 0.15, *p* =.046) and self-efficacy (β = 0.21, *p* =.010) were significantly associated with trust in Instagram. Self-efficacy was significantly associated with trust in television commercials (β = 0.26, *p* =.009), and trust in religious leaders, pastors, and preachers (β = 0.20, *p* =.048).

## Discussion

In this survey of 206 SGM adolescents in Alabama, we documented levels of trust in HIV prevention information about PrEP from different types of people and sources. We identified significant differences in reported levels of trust across race, ethnicity, and gender identity. Following adjustment for demographics and other characteristics, social support was significantly associated with higher reported trust in healthcare professionals and family members. Self-efficacy was significantly associated with higher reported trust in Instagram, television commercials, and straight friends and peers. Enhanced understanding of where adolescents find trusted information and what factors are correlated with trust in different people and information sources may facilitate development of interventions designed to reduce risk of HIV through education.

There were notable differences across race, ethnicity, and gender in reported level of trust in different people and sources of information. Notably, trust in healthcare providers was markedly high across all groups. However, White respondents and transgender + GNC respondents reported significantly higher levels of trust as compared to Black and cisgender peers respectively. In contrast, there were consistent reports of low trust in politicians, though Black respondents reported significantly higher levels of trust than White respondents and transgender + GNC respondents reported significantly lower levels of trust than cisgender respondents. This is consistent with prior qualitative research suggesting that transgender adolescents interpret the credibility of health information in the context of the socio-political environment [17]. While most groups reported low levels of trust in religious leaders, Black respondents reported significantly higher trust than White respondents. This finding affirms efforts to incorporate religious leaders in HIV prevention efforts tailored for Black populations [27, 28]. Critically these comparisons illustrate that healthcare providers remained trusted post-COVID, but other types of people may serve as important conduits of information about PrEP if they are trusted by members of that community. Moreover, characteristics beyond demographic traits warrant consideration when developing any intervention for this population.

Self-efficacy has previously been reported with increased trust in internet-based health information as it is posited individuals with higher self-efficacy feel greater confidence in their ability to oversee their own health and discern the quality of health information provided via internet sources [29, 30]. Thus, our finding of higher levels of trust in Instagram among those with high reported self-efficacy may reflect this very confidence in their ability to delineate between accurate health information and misinformation or disinformation. Instagram is among the most commonly used social media sites by adolescents and thus, it may constitute a low-cost means of delivering HIV prevention messaging [31]. Previous work has illustrated its efficacy in reaching Latinx men who have sex with men (MSM) who experience elevated risk of HIV acquisition [32]. This population also reported high levels of internalized homophobia that may dissuade information-seeking from traditional sources of HIV education, further amplifying the utility of a social media-based approach to disseminating this information. Interventions designed to promote uptake of HIV testing through social media have also been efficacious, particularly when designed and delivered in collaboration with community members [33–35]. These studies, however, also emphasized the importance of establishing a relationship through these platforms, which is more easily facilitated through social media sites like Facebook and Instagram than through platforms like YouTube or Twitter [36, 37]. Thus, while self-efficacy was an important predictor of trust in information obtained through Instagram, the role of social support in disseminating that information or promoting engagement with the health information may be critical to a social media-based intervention’s success.

Social support was identified as a significant predictor of increased levels of trust in healthcare providers and family members. Previous work has illustrated that higher levels of social support manifest in both higher levels of trust in health information from family members and increased health-information seeking [38]. This finding also comports with the hypothesis that social media interventions are most effective when delivered in such a way that promotes relationship-building [32, 36, 38, 39]. Exploration of social support and health-information seeking in SGM communities suggested that members social support networks were the primary source of health information [40]. For SGM adolescents, their social support may consist of family members, peers, and online communities [15], explaining the finding of higher levels of trust in family members among those with high levels of social support. Additionally, it is plausible that SGM adolescents with high levels of social support self-select to receive care from affirming healthcare providers when available, thereby increasing their trust in health information delivered by those healthcare providers. Conversely, those adolescents with lower social support may not have access to the same volume or quality of information that can facilitate their healthcare decision-making and elevate their trust in healthcare providers. As adolescents increasingly find community and social support in online spaces, ensuring that online interventions contain a component of relationship-building may promote trust in healthcare providers and, ultimately, uptake of HIV/STI testing and prevention. It is important to note that there were no significant predictors for trust in straight friends and peers, suggesting that such efforts to promote social support and relationship-building may be best suited to center SGM adolescents.

There are limitations to this study. While these data constitute an important and novel contribution to our understanding of trust in health information delivery among SGM males, the small sample size precluded more sophisticated analyses. All respondents received the same survey directions, but respondents were recruited both in-person and online. As such, there may have been differences in how the survey was read or interpreted. The recruitment approach, including recruitment from an LGBTQ-supportive school, limits the ability to generalize these results to broader populations of SGM adolescents in the southern US. While we recruited 206 SGM males, the number of respondents who reported a race other than White or African American/Black precluded robust analyses. Moreover, 32% of Hispanic respondents declined to report a race, limiting our ability to examine the race and ethnicity in combination. Some of the combinations, such as African/American Black and Hispanic, were also reported by fewer than ten respondents, leading to concerns about the ability to protect privacy and confidentiality. Additionally, the sample size of TGD respondents was quite small, perhaps limiting generalizability to the broader population of TGD adolescents in Alabama. Future work may oversample these sub-populations to permit more granular understanding of their trust in people and information sources. Despite limitations, this work constitutes novel understanding of which factors operate to potentially influence trust in different sources of information among highly vulnerable SGM adolescents.

In this survey of SGM adolescents in Alabama, we documented significant differences in levels of reported trust in information about PrEP by source of that information. Upon modeling, both self-efficacy and social support were identified as significant predictors of trust in different sources of information. These data may subsequently inform intervention development designed to increase PrEP uptake through information delivery by trusted sources.

## Data Availability

The datasets generated during and/or analyzed during the current study are not publicly available due to concerns about individual privacy. Data may be available from the corresponding author on reasonable request.

## Abbreviations

SGM: Sexual and gender minority
HIV: Human immunodeficiency virus
LGBTQ: Lesbian, gay, bisexual, transgender, and queer
SD: Standard deviation
STI: Sexually transmitted infection
US: United States
PrEP: Pre-exposure prophylaxis
MMSC: Male-to-male sexual contact
SAM-KAP: Survey of Adolescent MSM Knowledge and Preferences
GNC: Gender non-conforming
MSM: Men who have sex with men

## Footnotes

1 For analytical purposes, we chose the trust information sources that were shown to be different across race, gender, or ethnicity groups.
